# The impact of a child’s inborn error of metabolism: the parents’ perspectives on restrictions, discrimination, family planning, and emergency management

**DOI:** 10.1186/s13023-024-03315-6

**Published:** 2024-08-26

**Authors:** Tanjana Harings, Martina P. Neininger, Simone Eisenhofer, Alena G. Thiele, Wieland Kiess, Astrid Bertsche, Thilo Bertsche, Skadi Beblo

**Affiliations:** 1 Clinical Pharmacy, Institute of Pharmacy, Medical Faculty, Leipzig University, and Drug Safety Center, Leipzig University and Leipzig University Hospital, Bruederstrasse 32, 04103 Leipzig, Germany; 2 Center for Pediatric Research, University Hospital for Children and Adolescents, Liebigstrasse 20a, 04103 Leipzig, Germany; 3 Division of Neuropediatrics, University Hospital for Children and Adolescents, Ferdinand-Sauerbruch-Strasse 1, 17475 Greifswald, Germany; 4 Center for Rare Diseases, Leipzig University Medical Center, Philipp-Rosenthal-Strasse 55, 04103 Leipzig, Germany

**Keywords:** Inborn errors of metabolism, Restrictions, Family planning, Metabolic emergency management, Parents’ perceptions

## Abstract

**Background:**

To investigate the impact of children’s inborn error of metabolism (IEMs) on the children’s and their parents’ lives from the parents’ perspective. We focused on disease-related restrictions in various issues of daily life, experienced discrimination, parental family planning, and management of metabolic emergencies.

**Methods:**

We conducted a questionnaire-based survey with 108 parents of 119 children with IEM who attended a metabolic outpatient clinic. The children were categorized into 4 cohorts, based on increasing disease severity (cohort 1: IEMs with lowest severity, cohort 4: IEMs with highest severity), and compared by using Tobit regressions.

**Results:**

The severity of the child’s IEM was associated with an increase in the intensity of perceived restrictions from the parents’ perspective for themselves and their children in all aspects of life: in general, in contact with friends, in the pursuit of hobbies, in childcare/school/occupation, and due to emotional stress. The highest intensity of restrictions in all cohorts was found for the parents themselves in contact with friends (compared to cohort 1: cohort 2: c. 3.556, *p* = 0.002; cohort 3: c. 4.159, *p* = 0.003; cohort 4: c. 7.224, *p* < 0.001). Parents of 8% of children reported that their children were discriminated against because of IEM, with the highest proportion of affected children (43%) in cohort 4. Parental family planning decisions were influenced in 34% of parents, with fear of recurrence being a predominant aspect. Of the parents of children diagnosed with IEMs associated with metabolic emergencies, 68% stated that they felt well or very well prepared for the occurrence of a metabolic emergency, and 100% of parents were able to name the necessary action steps from memory. Nevertheless, 58% stated that they experienced an occurring emergency as rather or very stressful.

**Conclusions:**

From the parents’ perspective, the intensity of restrictions increased with the severity of the child’s IEM. The study shows the high impact of IEM on parents of children with IEM and the daily challenges they face. These findings emphasize the importance of comprehensive support for parents of children with IEM.

**Supplementary Information:**

The online version contains supplementary material available at 10.1186/s13023-024-03315-6.

## Background

Inborn errors of metabolism (IEMs) are a group of genetic disorders which significantly affect the body’s normal metabolic processes. With approximately one in 500 newborns worldwide affected by one of the up to 1904 different known IEMs [1–5], early diagnosis and rapid initiation of therapy are critical to patient prognosis and survival [6]. Nationwide newborn screening was introduced in many countries, including Germany, as early as 1968 to detect certain treatable metabolic disorders, such as phenylketonuria (PKU) [1, 7]. This has undoubtedly improved life expectancy and health outcomes for those affected [8, 9].

Existing studies have focused primarily on PKU, which is considered a model disease, and have often examined the quality of life of those affected and their families [10–14]. Although medical advances and increased life expectancy have benefited patients, there is still little scientific literature on the impact of the condition on daily lives of affected children and families, and the restrictions they experience. There is also a paucity of literature on how IEM affects parents’ family planning decisions.

A metabolic emergency can manifest itself clinically in various ways, but it can often be life-threatening. Effective management of metabolic emergencies is essential to prevent serious complications such as hepatic encephalopathy, or growth and mental retardation, or even death [15, 16].

Our study aimed at comprehensively investigating the impact of the child’s IEM on the affected children and parents from the parents’ perspective, particularly with regard to restrictions in various issues of daily life and on parental family planning. We also investigated parents’ perceptions and parental management of metabolic emergencies.

## Methods

### Patients and setting

In this exploratory cross-sectional survey, we consecutively invited all parents of children who had an appointment at the pediatric outpatient clinic for metabolic disorders of a university hospital between May 2020 and September 2020 to participate. The children were diagnosed with IEM or 3 other diseases leading to severe hypoglycemia, namely congenital hyperinsulinism, Beckwith-Wiedemann syndrome, or ketotic hypoglycemia. Parents with more than one affected child were asked to answer the questions in the survey for each eligible child, but only once for the questions concerning the parents themselves. The study was approved by the responsible ethics committee. Written informed consent was obtained from all participating parents, who had to be the legal guardians of the children.

Since the term IEM covers a wide spectrum of diseases of varying severity, the children were divided into four cohorts according to the severity of their disease. To compare the subjective restrictions mentioned by the parents, the classification into cohorts was done by specialists from the metabolic outpatient clinic of the university hospital to ensure an objective, medical basis (Table 1). Disease severity was classified on a patient-by-patient basis, assuming constant medical care and adherence to therapy. This means that the same diagnoses can occur in different cohorts, as the individual health status of the children and the time of diagnosis or start of treatment were considered. To determine possible differences in the intensity of the restrictions between IEM with and without metabolic emergencies, the cohorts were again divided into sub-cohorts.

**Table 1:** Characteristics of children and parents

Characteristics	Children (*n* = 119)	Parents (*n* = 108)
Median age (Q25/Q75; min/max) [years]	8 (3.5/13; 0/17)	38 (34/44; 24/54)
Gender [n (%)]		
Male	72 (61)	16 (15)
Female	47 (39)	92 (85)
Education [n (%)]		
Not yet in childcare	13 (11)	-
Childcare	36 (30)	-
Primary school	21 (18)	-
Middle school	12 (10)	54 (50)
Grammar school	23 (19)	53 (49)
Special needs school	8 (7)	-
Vocational training	6 (5)	-
No school degree	-	1 (1)
Professional education [n (%)]		
University/university of applied sciences degree	-	32 (30)
Completed vocational training	-	66 (61)
Other	-	7 (6)
No professional education	-	3 (3)
Working in healthcare [n (%)]	-	38 (35)
Metabolic emergency possible [n (%)]	47 (39)	47 (43)
Metabolic emergency already experienced [n (%)]	33 (28)	33 (31)
Diagnosis [n (%)]		
Cohort 1: With constant care and adherence, no complications or long-term damage are to be expected.	40 (34)	33 (31)
Biotinidase deficiency	5	4
Carnitine transporter deficiency	6	6
Familial hypercholesterolemia	18	14
Hyperphenylalaninemia	4	4
Unexplained hypoglycemia^a^	3	3
Isovaleric acidemia^a^	1	1
Unspecific disturbance of leucine metabolism^a^	3	1
Cohort 2: Despite constant care and adherence, complications or long-term damage are possible.	53 (45)	50 (46)
Beckwith-Wiedemann syndrome^a^	1	1
Glycogenosis type IX	1	1
Unexplained hypoglycemia^a^	2	2
Isovaleric acidemia^a^	1	1
Medium-chain-acyl-CoA-dehydrogenase-deficiency^a^	15	15
3-Methylcrotonyl-CoA-carboxylase deficiency^a^	1	1
Methylenetetrahydrofolate-reductase-deficiency	2	2
Phenylketonuria	26	24
Severe neonatal vitamin B12 deficiency	2	1
Very-long-chain-acyl-CoA-dehydrogenase-deficiency^a^	2	2
Cohort 3: Despite constant care and adherence, complications or long-term damage to be expected.	12 (10)	12 (11)
6-Pyruvoyl-tetrahydropterin synthase deficiency	1	1
Galactosemia	1	1
Glutaraciduria type I^a^	1	1
Glycogenosis type I^a^	3	3
Congenital hyperinsulinism^a^	1	1
Isovaleric acidemia^a^	2	2
Long-chain-3-hydroxyacyl-CoA-dehydrogenase-deficiency^a^	2	2
Tyrosinemia type I^a^	1	1
Cohort 4: Despite constant care and adherence, uncorrectable complications are acutely present.	14 (12)	13 (12)
α-Mannosidosis	1	1
Ketotic hypoglycemia^a^	1	1
Non-ketotic hyperglycinemia^a^	1	1
Diazoxide-sensitive hyperinsulinism^a^	1	1
Long-chain-3-hydroxyacyl-CoA-dehydrogenase-deficiency^a^	1	1
Morbus Niemann-Pick type A	1	1
Ornithine-transcarbamylase deficiency^a^	1	1
Pyruvate dehydrogenase deficiency^a^	1	1
Sepiapterin reductase deficiency	2	1
Smith-Lemli-Opitz syndrome^b^	3	3
X-Adrenoleukodystrophy^a^	1	1

^a^Condition with potential metabolic emergency

^b^One child was also diagnosed with Medium-chain-acyl-CoA-dehydrogenase deficiency^a^

Disease severity was classified on a patient-by-patient basis, assuming constant medical care and adherence to therapy. This means that the same diagnoses can occur in different cohorts, as the individual health status of the children and the time of diagnosis or start of treatment were considered. Children whose exact disease was not known at the time of the study were also assigned to a cohort based on their symptoms and health status

### Structured interview

After written consent to participate, the interview was conducted by telephone based on a questionnaire consisting of predefined questions (Additional File 1). The following issues were addressed:

1. Restrictions of children and parents in various issues of daily life due to the child’s IEM.
2. Discrimination of children due to their IEM.
3. Impact of the child’s IEM on parental family planning.
4. Management of metabolic emergencies (if applicable).
5. Sociodemographic data of children and parents.

The questionnaire was pretested to ensure comprehensibility, and adjustments were made to the questions based on the received feedback. The parents’ perceptions and the experienced intensity of various constraints were evaluated using Likert scales ranging from 0 to 5 (0: not at all, 5: very often/very much/very well/very severe). To assess the emergency management, typical symptoms of a potential metabolic emergency were described to the parents (Additional File 1) and they were asked to describe how they would act in such a situation. The parents’ responses were compared with the action steps described in emergency plans provided by the treating physician and evaluated by a panel of experts. To facilitate quantitative analysis, responses to open questions were clustered by the interdisciplinary study team consisting of pediatricians (including those specialized in IEM), pharmacists and nutritionists.

### Analysis and statistics

Calculations were performed using SPSS (Statistical Package for the Social Science, Version 29.0.1.0, IBM, Armonk, New York, USA) and Eviews (Version, HIS Global Inc., Irvine, California, USA). Frequencies are reported as numbers and percentages, continuous data as median with first and third quartile (Q25/Q75) and range (min/max). For comparisons between the cohorts, we applied chi-square tests, Fisher’s exact tests, Kruskal-Wallis tests, Mann-Whitney-U tests, or Tobit regressions, depending on the underlying data. For the regressions, we report coefficients (c.) and *p*-values. A *p-*value ≤ 0.05 was considered to indicate significance. When multiple testing (*n* = 6) for the comparison of the cohorts was necessary, we applied the Bonferroni correction and considered an adjusted *p*-value ≤ 0.008 as significant.

## Results

### Characteristics of children and parents

Throughout the study period, 152 parents were invited to participate of whom 118 (76%) agreed to take part. Ten parents had to be excluded either because they could not be contacted at the appointed interview date (6/152, 4%) or they withdrew their agreement (4/152, 3%). Thus, 108 parents of 119 children were enrolled (Table 1). Of the children, 47/119 (39%) were diagnosed with IEMs associated with potential metabolic emergencies.

### Restrictions of children and parents in various issues of daily life due to the child’s IEM

**Fig. 1:**
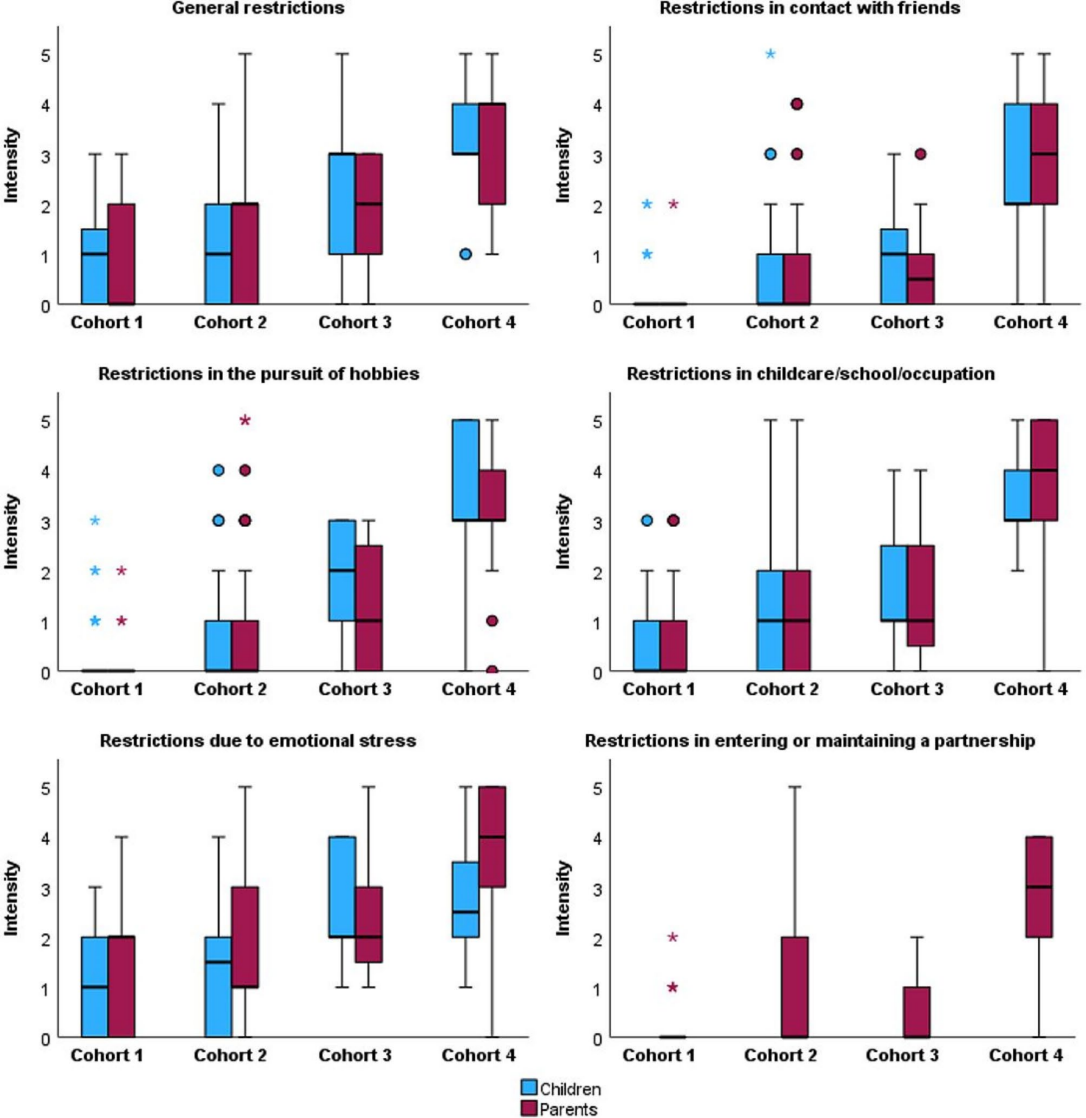
Title: Intensity of restrictions on daily life for children and parents from the parent’s perspective. Legend: Scale ranging from 0 = No restrictions at all to 5 = Very severe restrictions. Cohort 1: With constant care and adherence, no complications or long-term damage are to be expected. Cohort 2: Despite constant care and adherence, complications or long-term damage are possible. Cohort 3: Despite constant care and adherence, complications or long-term damage to be expected. Cohort 4: Despite constant care and adherence, uncorrectable complications are acutely present. The restrictions in entering or maintaining a partnership were only surveyed for the parents

The intensity of restrictions of children and parents due to the children’s IEMs in various issues of daily life from the parents’ perspective is shown in Fig. 1. The comparison of cohort 1 with cohort 2, cohort 3, and cohort 4 showed that the intensity of restrictions increased with increasing severity of IEM, both for children and parents. Compared to cohort 1, the parents reported an increase in the severity of general restrictions for children (cohort 2: c. 0.849, *p* = 0.019; cohort 3: c. 2.012, *p* < 0.001; cohort 4: c. 3.230, *p* < 0.001), as well as for themselves (cohort 2: c. 1.467, *p* = 0.001; cohort 3: c. 1.939, *p* = 0.002; cohort 4: c. 3.636, *p* < 0.001). For cohort 4, the greatest restrictions were mentioned in every issue of daily life for both the children and the parents compared to cohort 1 (each issue of daily life: *p* < 0.001). Parents were also asked about the financial burden due to their children’s IEMs. Here too, the burden increased with increasing severity of IEM compared to cohort 1 (cohort 2: c. 1.783, *p* = 0.004; cohort 3: c. 2.012, *p* = 0.020; cohort 4: c. 3.178, *p* < 0.001). The intensity of restrictions on entering into or maintaining a partnership was only surveyed for the parents. Again, the parents of children in cohort 4 indicated the most intensive restrictions (c. 4.983, *p* < 0.001). Further details can be found in Additional File 2. Due to the very small number of patients per cohort, it was not possible to perform statistical tests to compare patients with IEM with potential metabolic emergencies and those without emergencies. However, the graphical analysis showed a similar trend in both sub-cohorts compared to the results of the main cohorts (Additional file 3).

Some parents felt the need to explain their assessments of restrictions. According to those parents, the main challenges for 10 children were dietary restrictions and for 3 children each difficulty with medication or the effect of the IEM on physical performance. For 4 parents, the reason given for the restrictions was the time needed to attend medical appointments due to long distances and time off work.

### Discrimination of children due to their IEM

Parents of 10/119 children (8%) reported that their children were discriminated against because of their IEMs. The sources of discrimination were other children (5/10), caregivers such as teachers and educators (5/10), family members (2/10), and society in general (2/10), as perceived by the parents. In cohort 2, discrimination was reported for 2/53 children, in cohort 3 for 2/12, and in cohort 4 for 6/14. The affected children had a median age of 5 years (Q25/Q75; min/max: 5/9.5; 1/17). Of the parents who reported discrimination, one child had a migrant background. Due to acute damage caused by the IEM, this child was mentally and physically impaired and dependent on a wheelchair. For the sibling who had the same diagnosis but was not dependent on a wheelchair, the parents did not report any discrimination.

### Impact of the child’s IEM on parental family planning

An influence on parental family planning due to their child’s IEM was reported by 37/108 (34%) parents. In detail, 23/108 (21%) did not want another child due to the risk of recurrence of IEM, 11/108 (10%) were unsure about having another child, 2/108 (2%) considered prenatal diagnostics, and 1/108 (1%) did not specify the type of influence.

The influence on parental family planning rose with increasing severity of their child’s IEM. Of the parents of children in cohort 1 2/33 reported an influence, 19/50 in cohort 2, 6/12 in cohort 3 and 10/13 in cohort 4. There were significant differences between the following cohorts: 1–2 (*p* < 0.001), 1–3 (*p* = 0.001), 1–4 (*p* < 0.001) and 2–4 (*p* = 0.006).

### Management of metabolic emergencies

In the described scenarios for typical symptoms of a metabolic emergency, the parents of all (47/47) children who had been diagnosed with IEM associated with potential metabolic emergencies replied adequately and named exactly the action steps as described in the emergency plans provided by the attending physicians to the families. The parents of 34/47 children responded that they had a written emergency plan, and 16/47 stated that they always carried the plan with them. The remaining parents stated that they received verbal instructions from the physician.

Parental perceptions of metabolic emergencies did not significantly differ between cohorts or between parents with or without experience of metabolic emergencies (Table 2). Of the parents, 32/47 stated that they felt well or very well prepared for the occurrence of a metabolic emergency. Nevertheless, 18/31 of the parents who had already experienced metabolic emergencies reported that those situations had been rather or very stressful for them.

**Table 2:** Parental perceptions of metabolic emergencies based on severity of the child’s IEM or previous experience

	Severity of the child’s IEM				Experience with metabolic emergency	
	Cohort 1 (*n* = 7)	Cohort 2 (*n* = 22)	Cohort 3 (*n* = 10)	Cohort 4 (*n* = 8)	yes (*n* = 33)	no (*n* = 14)
How often do you think about the occurrence of a metabolic emergency?	3 (1/3; 1/3)	1 (0/2; 0/5)	2 (1/2; 1/3)	2 (1/2; 1/3)	2 (1/2; 0/5)	1 (0/3; 0/3)
How much do you worry about metabolic emergencies?	3 (1/3; 0/3)	1 (1/3; 0/5)	3 (2/4; 1/5)	2 (1,5/3; 1/4)	3 (1/3; 0/5)	2 (1/3; 0/4)
How stressful did you experience the occurrence of a metabolic emergency? *	3.5 (2/4,5; 1/5)	4 (3/5; 0/5)	4 (3/5; 2/5)	3.5 (2/5; 1/5)	4 (3/5; 0/5)	-
How well prepared do you feel for the occurrence of a metabolic emergency?	5 (4/5; 3/5)	5 (3/5; 2/5)	4 (3/4; 2/5)	4 (3/4; 2/5)	4 (4/5; 2/5)	3.5 (3/5; 2/5)

Likert scale ranging from 0 = not at all to 5 = very often/very much/very well. The median value (Q25/Q75; min/max) is presented

*Question only asked in parents, who already experienced metabolic emergencies

Cohort 1: With constant care and adherence, no complications or long-term damage are to be expected

Cohort 2: Despite constant care and adherence, complications or long-term damage are possible

Cohort 3: Despite constant care and adherence, complications or long-term damage to be expected

Cohort 4: Despite constant care and adherence, uncorrectable complications are acutely present

## Discussion

The study aimed to assess the impact of IEM on the lives of children and their parents from the parents’ perspective. With increasing severity of IEMs, the parents reported more severe restrictions in all issues of daily life. Additionally, the proportion of children who experienced discrimination, according to the parents, was the highest in the cohort with the highest severity of IEM. One-third of the parents reported that further family planning was influenced by the children’s IEMs. All parents were able to name the necessary action steps that are required when symptoms of a metabolic emergency occur. A particular strength of the study is that it was not limited to a single IEM, but rather considered a wide range of IEMs, shedding light on under-researched patient populations. In these often very rare or very severe IEMs, research is limited due to small patient numbers or early mortality. As we included those patients, cohort 3 and cohort 4 are relatively small compared to cohort 1 and cohort 2. This may have had an impact on the validity of the results from cohort 3 and cohort 4, which may limit their generalizability.

### Restrictions of children and parents in various issues of daily life due to the child’s IEM

In our study, increasing severity of IEM was associated with more restrictions in various issues of daily life for both children and parents, according to the parents. This finding is in line with previous studies that showed an impact of disease severity on the psychological well-being of children with IEM and their families [17]. Families of children with other conditions, e.g. with epilepsy/developmental disorders, also reported more severe restrictions with increasing severity of the condition [18].

The parents of cohort 2 and cohort 4 stated severe restrictions in childcare/school/occupation in some cases, often due to the nutritional demands of the IEM. Despite advancements in dietary therapy, nutritional restrictions remained a major burden for children as also described in other studies [19–22].

It has been shown that children with IEM have higher levels of emotional problems, particularly with increasing severity of the disease [10, 17]. Interestingly, parents in our study often reported similar or even the same restrictions regarding emotional stress for themselves as for their children, as well as in all other issues of daily life. It is known from other studies that mothers are particularly affected by restrictions, as they usually take on a large part of the care work for chronically ill children [23]. The high proportion of mothers participating in our study emphasizes this. Due to many challenges for parents of affected children, the care of children with IEM requires different types of support, e.g. psychological or financial [24–26]. Although the physical, intellectual, and behavioral impairments can be partially mitigated by early diagnosis due to newborn screening, the diagnosis of IEM places an enormous emotional burden on parents [27]. Other studies showed that sometimes the perceived parental burden of the children’s disease is greater than that of the patients themselves [13, 18, 27, 28]. One reason for this could be that there is a kind of “before/after” for the parents, namely separated by the day on which the diagnosis was made. For the children, on the other hand, there is usually no “before”, so it might be difficult for the parents to imagine what it could have been like, and therefore the potential loss might not be experienced as strongly. This underlines the importance that not only the affected children need support from healthcare professionals but also their parents [29].

Many children with IEM require special diets for the treatment of the specific IEM. These diets are typically four to ten times more expensive than conventional foods and often not covered by health insurances [30]. In our study, the parents of children in cohort 4, who were often mentally and/or physically impaired due to their IEM, reported an increased financial burden. The intensive care requirements of children with rare diseases such as IEM can be accompanied by increased costs in addition to special diets, e.g. for special treatments or medication [29]. Furthermore, costs may also arise in the context of a barrier-free conversion of the living space or due to (medical) aids for the care of the children [29].

The division into sub-cohorts of patients with IEM with and without metabolic emergencies showed similar results to the main cohorts. It could therefore be assumed that it is of secondary importance for parents in everyday life whether acute damage can occur now or in the future. Due to the heterogeneity of the individual IEMs, it cannot be determined from the results whether it is the restrictive therapy, as in patients with PKU, or the potential for metabolic emergencies, as in patients with Medium-chain-acyl-CoA-dehydrogenase-deficiency, that restricts parents the most.

### Discrimination of children due to their IEM

Less than 10% of the parents stated that their children were discriminated against due to their IEM. In another study on pediatric patients with epilepsy and/or developmental disorders, 47% of the parents reported that their children were discriminated against, mainly by other children [18]. In both, the epilepsy study and our study, the same perpetrators of discrimination were mentioned. One possible explanation for the relatively rare occurrence of discrimination in our patient collective could be that epilepsy and developmental disorders can be more visible and are more likely to be noticed by others than most IEMs if diagnosed early and treated in time. Furthermore, emergencies in patients with epilepsy might seem more threatening because of the seizures than those in IEMs, which are usually accompanied by nausea, vomiting or sudden lack of consciousness. Nevertheless, the proportion of children who experienced discrimination due to IEM increased with the severity of the IEM, likely due to more visible physical or mental impairments.

### Impact of the child’s IEM on parental family planning

One-third of parents with children with IEM reported their future family planning was influenced by their child’s IEM, mainly by fear of recurrence of the IEM. The proportion of parents reporting an impact on family planning increased with the severity of IEM. In a study about reproductive decisions in families affected by IEMs, it was shown that 41% had taken steps to prevent a future pregnancy, 56% would consider prenatal diagnosis, and 10% would terminate a pregnancy affected by an IEM [31]. These figures are similar to those reported in our study. From the study by Read [31], it appears that decisions were made primarily due to a lack of social support rather than a lack of advice from physicians and other professionals. The reasons for deciding against another pregnancy among parents in our study were not asked. However, it can be assumed that they are similar to those mentioned in the study by Read [31].

### Management of metabolic emergencies

Our study demonstrates that parents of children with IEM, who are cared for in a metabolic outpatient clinic, are highly knowledgeable about the condition. All parents were able to recall the physicians’ recommendations on action steps for typical symptoms of a metabolic emergency, which were described in the emergency plan. Nevertheless, the parents of only one-third of the children stated that they always carried the emergency plan with them. Regardless of the severity of the children’s underlying IEMs or their previous experiences with metabolic emergencies, parents understandably reported high levels of distress experienced in actual metabolic emergencies, as these situations are always life-threatening for the children. It is therefore important to support the parents mentally and emotionally.

## Conclusions

IEM severity increases challenges for parents of affected children, including managing dietary restrictions, emotional burden, and potential discrimination. Parents with severely affected children reported more frequently concerns about future pregnancies. This study highlights the importance of a comprehensive care, including emotional support, financial assistance, and genetic counselling.

### Electronic supplementary material

Below is the link to the electronic supplementary material.

Supplementary Material 1

Supplementary Material 2

Supplementary Material 3

## Data Availability

The dataset generated and analyzed during the current study are not publicly available due to ethical and privacy considerations to protect the confidentiality of participants but are available from the corresponding author on reasonable request.

## Abbreviations

IEM: Inborn error of metabolism
PKU: Phenylketonuria
