# Supplementary material for: The impact of a child’s inborn error of metabolism: the parents’ perspectives on restrictions, discrimination, family planning, and emergency management
Source: Orphanet J Rare Dis. 2024 Aug 26;19:313. doi: 10.1186/s13023-024-03315-6 (PMC11348755; doi:10.1186/s13023-024-03315-6)
Supplement: Supplementary file 1 — Supplementary Material 1 [file 13023_2024_3315_MOESM1_ESM.pdf]

Additional file 1: Questionnaire

The original questionnaire was in German. We provide a translation to facilitate comprehensibility.

1) **Restrictions of children and parents in various issues of daily life due to child's IEM**
   The following questions are about your assessment of your child's restrictions due to the inborn error of metabolism (IEM). You can answer on a scale ranging from 0 to 5. 0 corresponds to no restrictions at all, 5 to very severe restrictions. There is no right or wrong answer to these questions. Please answer spontaneously. It may be that some questions are not yet relevant for your child, for example due to age. In this case, just let me know.
   i. On a scale ranging from 0 to 5, how would you rate your child's general restrictions due to the IEM?
   ii. On a scale ranging from 0 to 5, how would you rate your child's restrictions in contact with friends due to the IEM?
   iii. On a scale ranging from 0 to 5, how would you rate your child's restrictions on the pursuit of hobbies due to the IEM?
   iv. On a scale ranging from 0 to 5, how would you rate your child's restrictions at childcare/school/occupation due to the IEM?
   v. On a scale ranging from 0 to 5, how would you rate your child's restrictions due to emotional stress due to the IEM?

   The following questions are about your assessment of your own restrictions due to your child's IEM. Again, you can answer on a scale ranging from 0 to 5. 0 corresponds to no restrictions at all, 5 to very severe restrictions.

   i. On a scale ranging from 0 to 5, how would you rate your general restrictions due to your child's IEM?
   ii. On a scale ranging from 0 to 5, how would you rate your restrictions in contact with friends due to your child's IEM?
   iii. On a scale ranging from 0 to 5, how would you rate your restrictions in practicing hobbies due to your child's IEM?
   iv. On a scale ranging from 0 to 5, how would you rate your restrictions in your professional life due to your child's IEM?
   v. On a scale ranging from 0 to 5, how would you rate your restrictions due to emotional stress due to your child's IEM?
   vi. On a scale ranging from 0 to 5, how would you rate your restrictions in entering into or maintaining a partnership due to your child's IEM?

2) **Discrimination of children due to their IEM**
   i. Do you feel that your child is discriminated against because of the IEM?
   ii. [If yes] To what extent and by whom?

3) **Impact of child's IEM on parental family planning**
   i. Has your child's IEM influenced your family planning?
   ii. [If yes] In which way?

4) Management of metabolic emergencies

i. Is your child’s IEM associated with potential metabolic emergencies?
ii. [If yes] I will now present you with a scenario. Please imagine the situation and tell me how you would act. Please imagine that your child has a mild infection with some diarrhea and occasional vomiting [for glycogenosis type 1A: the blood glucose level is around 4 mmol/L]. What do you think of in this situation in relation to your child's metabolic disease and how would you proceed?
iii. Now imagine your child has a fever, repeated diarrhea or vomits regularly [For glycogenosis type 1A: Your child's blood glucose level is around 3.3 mmol/L]. How would you proceed now? [Additionally for glycogenosis type 1A: When would you call an ambulance?]
iv. Has a metabolic emergency ever occurred?
v. Do you have an emergency plan for your child?
vi. [If yes] How often do you usually carry this emergency plan with you?

Now I would like to ask you 4 more questions about your perception of metabolic emergencies. Again, you can answer on a scale ranging from 0 to 5. 0 means not at all, 5 means very often/very stressful/very good.

i. On a scale ranging from 0 to 5, how often do you think about the occurrence of a metabolic emergency?
ii. On a scale ranging from 0 to 5, how much do you worry about metabolic emergencies?
iii. [Only in parents who had already experienced a metabolic emergency in their children] On a scale ranging from 0 to 5, how stressful did you experience the occurrence of a metabolic emergency?
iv. On a scale ranging from 0 to 5, how well prepared do you feel for the occurrence of a metabolic emergency?

5) Sociodemographic data

i. How old is your child?
ii. Which gender is your child?
iii. Childcare/school/occupation
   a. Not yet in childcare
   b. Childcare
   c. Primary school
   d. Middle school
   e. Grammar school
   f. Special needs school
   g. Vocational preparation year
   h. Vocational training
   i. Other

iv. How old are you?
v. Which gender are you?
vi. What is your highest school degree?
   a. Primary school
   b. Middle school
   c. Grammar school
   d. Special needs school
   e. Other
   f. No school degree

vii. What is your highest professional qualification?
   a. University/University of applied sciences degree
   b. Completed vocational training
   c. Other
   d. No professional qualification

viii. Do you work in a healthcare profession?
