# Supplementary material for: The impact of a child’s inborn error of metabolism: the parents’ perspectives on restrictions, discrimination, family planning, and emergency management
Source: Orphanet J Rare Dis. 2024 Aug 26;19:313. doi: 10.1186/s13023-024-03315-6 (PMC11348755; doi:10.1186/s13023-024-03315-6)
Supplement: Supplementary file 2 — Supplementary Material 2 [file 13023_2024_3315_MOESM2_ESM.pdf]

Additional file 2: Intensity of restrictions in various issues of daily life of children and parents from the parents' perspective compared to cohort 1.

	Children					Parents					
	General restrictions	Restrictions in contact with friends	Restrictions in the pursuit of hobbies	Restrictions in childcare/ school/ occupation	Restrictions due to emotional stress	General restrictions	Restrictions in contact with friends	Restrictions in the pursuit of hobbies	Restrictions in occupation	Restrictions due to emotional stress	Restrictions in entering or maintaining a partnership
Cohort 2	c. 0.849<br>$p = 0.019$	c. 1.718<br>$p = 0.009$	c. 1.005<br>n.s.	c. 1.139<br>$p = 0.005$	c. 0.820<br>$p = 0.038$	c. 1.467<br>$p = 0.001$	c. 3.556<br>$p = 0.002$	c. 2.783<br>$p = 0.002$	c. 1.403<br>$p = 0.027$	c. 0.751<br>n.s.	c. 1.912<br>$p = 0.010$
Cohort 3	c. 2.012<br>$p < 0.001$	c. 2.675<br>$p = 0.003$	c. 2.825<br>$p < 0.001$	c. 2.141<br>$p < 0.001$	c. 2.114<br>$p = 0.001$	c. 1.939<br>$p = 0.002$	c. 4.159<br>$p = 0.003$	c. 4.168<br>$p < 0.001$	c. 2.174<br>$p = 0.017$	c. 1.583<br>$p = 0.008$	c. 1.541<br>n.s.
Cohort 4	c. 3.230<br>$p < 0.001$	c. 4.747<br>$p < 0.001$	c. 5.154<br>$p < 0.001$	c. 3.753<br>$p < 0.001$	c. 2.446<br>$p < 0.001$	c. 3.646<br>$p < 0.001$	c. 7.224<br>$p < 0.001$	c. 6.492<br>$p < 0.001$	c. 5.087<br>$p < 0.001$	c. 2.691<br>$p < 0.001$	c. 4.983<br>$p < 0.001$

Cohort 1: With constant care and adherence, no complications or long-term damage are to be expected.
Cohort 2: Despite constant care and adherence, complications or long-term damage are possible.
Cohort 3: Despite constant care and adherence, complications or long-term damage to be expected.
Cohort 4: Despite constant care and adherence, uncorrectable complications are acutely present.
n.s.: not significant
