# Supplementary material for: The impact of a child’s inborn error of metabolism: the parents’ perspectives on restrictions, discrimination, family planning, and emergency management
Source: Orphanet J Rare Dis. 2024 Aug 26;19:313. doi: 10.1186/s13023-024-03315-6 (PMC11348755; doi:10.1186/s13023-024-03315-6)
Supplement: Supplementary file 3 — Supplementary Material 3 [file 13023_2024_3315_MOESM3_ESM.pdf]

Additional file 3: Intensity of restrictions in various issues of daily life of children and parents from the perspective of parents of children with and without metabolic emergencies.

Legend: Scale ranging from 0 = No restrictions at all to 5 = Very severe restrictions.

Cohort 1: With constant care and adherence, no complications or long-term damage are to be expected.

Cohort 2: Despite constant care and adherence, complications or long-term damage are possible.

Cohort 3: Despite constant care and adherence, complications or long-term damage to be expected.

Cohort 4: Despite constant care and adherence, uncorrectable complications are acutely present. The restrictions in entering or maintaining a partnership were only surveyed for the parents.

Cohort	Metabolic emergencies	Parents n	Patients n
1	With possible metabolic emergencies	5	7
1	Without possible metabolic emergencies	28	33
2	With possible metabolic emergencies	22	22
2	Without possible metabolic emergencies	27	31
3	With possible metabolic emergencies	10	10
3	Without possible metabolic emergencies	2	2
4	With possible metabolic emergencies	8	8
4	Without possible metabolic emergencies	5	6

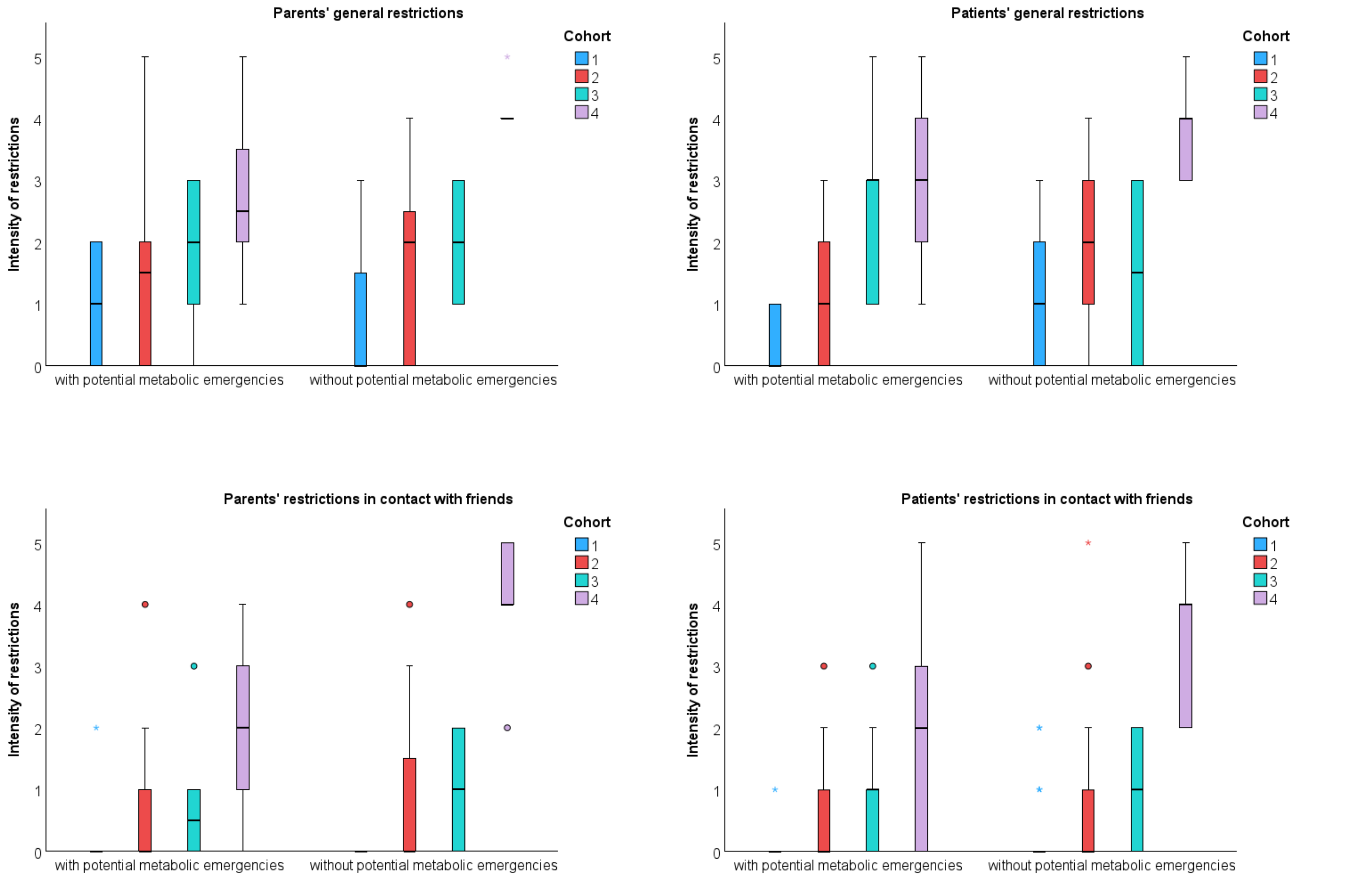
Parents' general restrictions
Patients' general restrictions
Parents' restrictions in contact with friends
Patients' restrictions in contact with friends
Intensity of restrictions
Cohort
1
2
3
4
0
1
2
3
4
5
with potential metabolic emergencies
without potential metabolic emergencies

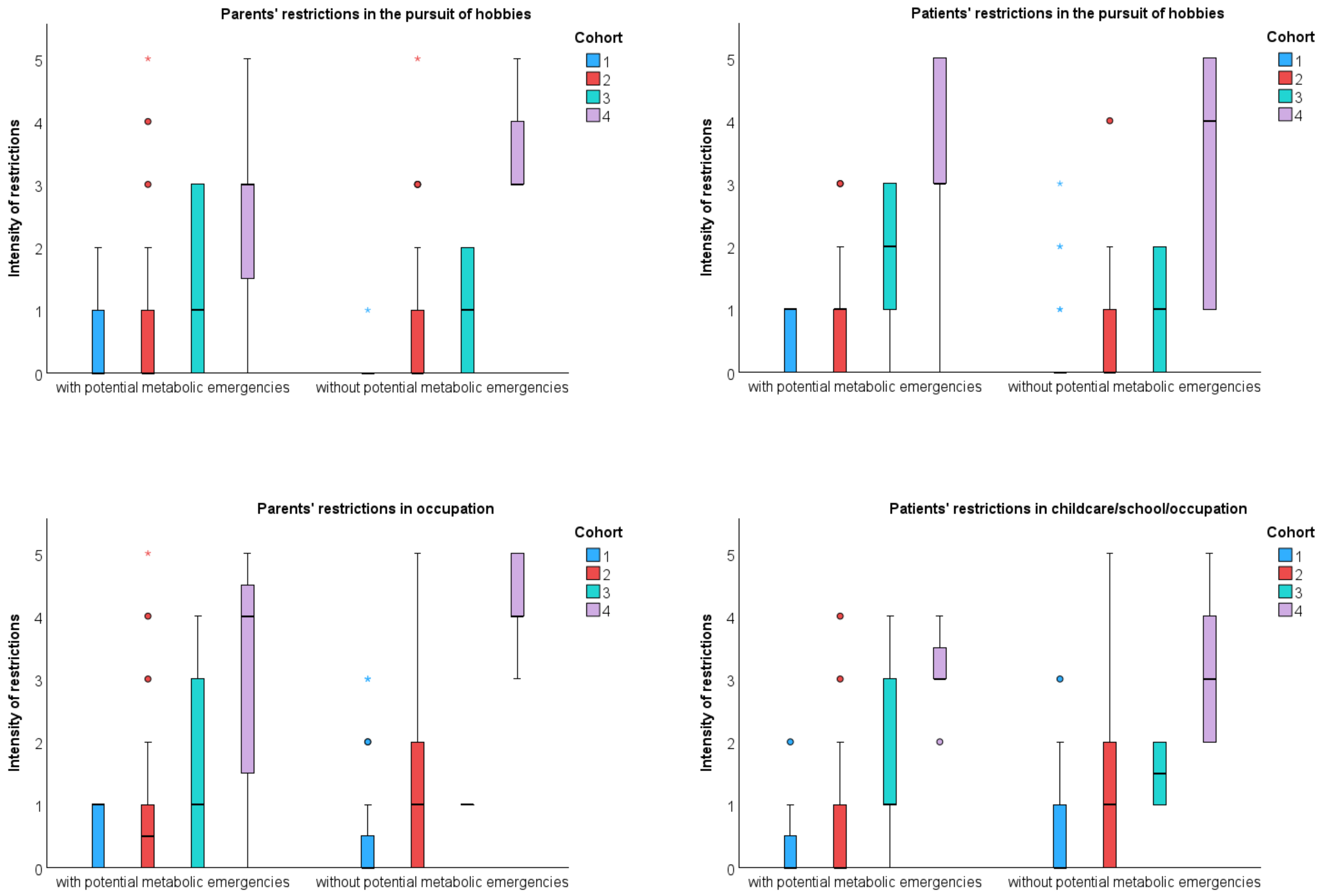

Parents' restrictions in the pursuit of hobbies
Patients' restrictions in the pursuit of hobbies
Parents' restrictions in occupation
Patients' restrictions in childcare/school/occupation
Intensity of restrictions
0
1
2
3
4
5
with potential metabolic emergencies
without potential metabolic emergencies
Cohort
1
2
3
4

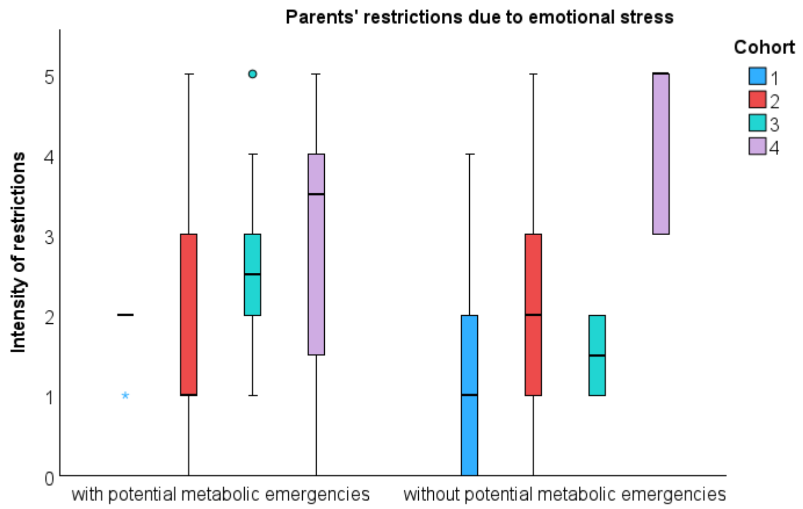

Parents' restrictions due to emotional stress
Intensity of restrictions
0
1
2
3
4
5
with potential metabolic emergencies
without potential metabolic emergencies
Cohort
1
2
3
4

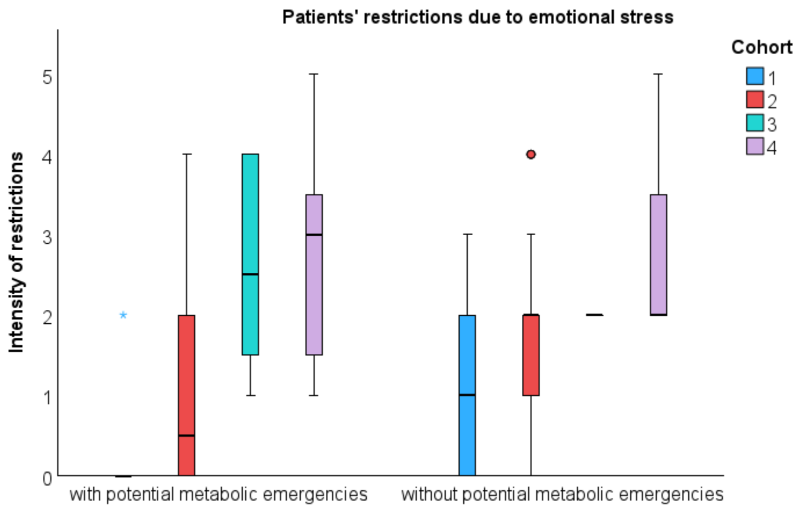

Patients' restrictions due to emotional stress
Intensity of restrictions
0
1
2
3
4
5
with potential metabolic emergencies
without potential metabolic emergencies
Cohort
1
2
3
4

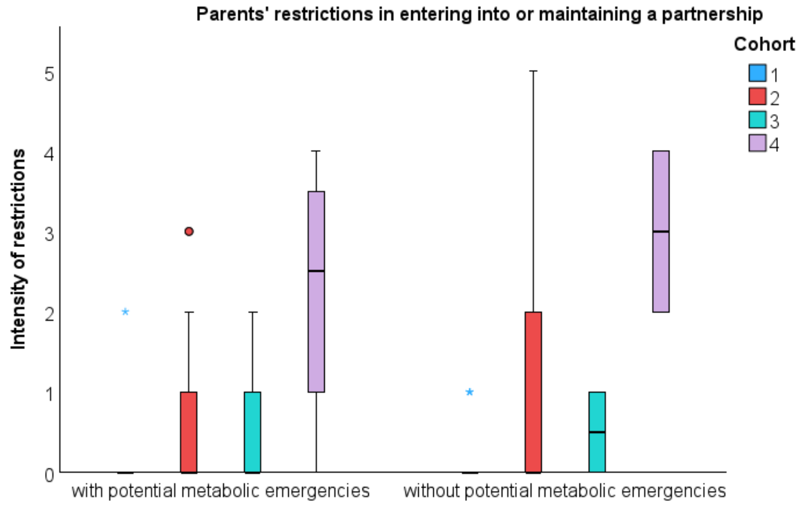

Parents' restrictions in entering into or maintaining a partnership
Intensity of restrictions
0
1
2
3
4
5
with potential metabolic emergencies
without potential metabolic emergencies
Cohort
1
2
3
4
